# Longitudinal Impact of Hurricane Sandy Exposure on Mental Health Symptoms

**DOI:** 10.3390/ijerph14090957

**Published:** 2017-08-24

**Authors:** Rebecca M. Schwartz, Christina N. Gillezeau, Bian Liu, Wil Lieberman-Cribbin, Emanuela Taioli

**Affiliations:** 1 Department of Occupational Medicine, Epidemiology and Prevention, Hofstra Northwell Health School of Medicine, Great Neck, NY 11021, USA; Rschwartz3@northwell.edu; 2 Department of Population Health Science and Policy and Institute for Translational Epidemiology, Icahn School of Medicine at Mount Sinai, New York, NY 10029, USA; cnanderson89@gmail.com (C.N.G.); bian.liu@mountsinai.org (B.L.); wil.lieberman-cribbin@icahn.mssm.edu (W.L.-C.)

**Keywords:** natural disasters, follow-up, anxiety, depression, post-traumatic stress disorder

## Abstract

Hurricane Sandy hit the eastern coast of the United States in October 2012, causing billions of dollars in damage and acute physical and mental health problems. The long-term mental health consequences of the storm and their predictors have not been studied. New York City and Long Island residents completed questionnaires regarding their initial Hurricane Sandy exposure and mental health symptoms at baseline and 1 year later (N = 130). There were statistically significant decreases in anxiety scores (mean difference = −0.33, *p* < 0.01) and post-traumatic stress disorder (PTSD) scores (mean difference = −1.98, *p* = 0.001) between baseline and follow-up. Experiencing a combination of personal and property damage was positively associated with long-term PTSD symptoms (OR_adj_ 1.2, 95% CI [1.1–1.4]) but not with anxiety or depression. Having anxiety, depression, or PTSD at baseline was a significant predictor of persistent anxiety (OR_adj_ 2.8 95% CI [1.1–6.8], depression (OR_adj_ 7.4 95% CI [2.3–24.1) and PTSD (OR_adj_ 4.1 95% CI [1.1–14.6]) at follow-up. Exposure to Hurricane Sandy has an impact on PTSD symptoms that persists over time. Given the likelihood of more frequent and intense hurricanes due to climate change, future hurricane recovery efforts must consider the long-term effects of hurricane exposure on mental health, especially on PTSD, when providing appropriate assistance and treatment.

## 1. Introduction

On 29 October 2012, Hurricane Sandy hit the Eastern Seaboard causing an estimated 71 billion dollars in damage [1], displacing 20,000 individuals from their homes, and directly causing the deaths of 117 people [2]. Additionally, Hurricane Sandy created a host of new hazards and challenges for those returning to their homes including limited access to gasoline, difficulty traveling due to the closure of and damage to the New York City subway system [3], and increased exposure to mold and other environmental toxins [4]. It is generally accepted that increased exposure to traumas are associated with increased stress and negative mental health outcomes, particularly post-traumatic stress disorder (PTSD) [5,6,7,8], although the social, environmental, and economic factors that exacerbate or mitigate these outcomes are not well understood. Additionally, most research has focused on the immediate aftermath of the disaster, while few studies have examined the long-term impact on mental health outcomes.

Research on Hurricane Katrina indicated that those with increased levels of exposure experienced worse mental and physical health at least one year after the disaster [9]. Although the number of stressors decreased over time among those exposed to Katrina, the psychological effects of those stressors persistently played a prominent role in people’s lives [10,11]. Results from Katrina cannot be generalized to all disasters, or even all hurricanes; although survivors of Hurricane Katrina were more likely to develop PTSD symptoms as a result of long-term property damage five years after the storm, as compared to Hurricane Andrew where PTSD symptoms among survivors were more likely to be associated with immediate, not long-term, hurricane exposures [12].

Certain populations are believed to be at increased risk of negative mental health symptoms after a disaster. After Hurricane Sandy, older adults were reported to be especially vulnerable, particularly if they lacked social support [13,14]. Additionally, research suggests that women and individuals who have a history of trauma, including experience coping with previous hurricanes and 9/11, may be at an increased risk of PTSD symptoms [6,15,16].

In recognition of the fact that disasters can create or exacerbate mental health issues, the Federal Emergency Management Agency (FEMA) allocated $50 million toward mental health services after Hurricane Sandy, although a 2015 study reported that 5.9% of exposed individuals still had unmet mental health needs [17]. A more targeted approach, however, is to connect individuals with mental health needs with additional resources, such as through Project Hope [18], although more research is required to predict and understand areas of future mental health needs.

Previous research has already established that increases in mental health disorders after Hurricane Sandy were clustered in geographic areas that were more exposed to the effects of the hurricane, and that within these clusters, minorities were at greater risk of PTSD symptoms [19,20]. However, despite the increase in mental health symptoms, there was a decrease in mental health-related visits to local emergency rooms in the month after Hurricane Sandy [21]. A 2015 study by Schwartz et al. [22] examined the relationship between hurricane exposures and level of perceived stress after Hurricane Sandy. This research reported that perceived stress was higher in areas more strongly affected by Hurricane Sandy, and that individual exposure was significantly associated with increased perceived stress levels [22]. In addition, our previous research indicated that increased exposure was associated with increased PTSD, depression, and anxiety symptoms approximately 1–2 years after the hurricane [23]. Building upon our previous studies, the current work assessed the long-term mental health status of a subgroup of respondents who completed mental health surveys at a second time point, one year after their first survey completion. We hypothesized that mental health symptoms persist over time and that initial Hurricane Sandy exposure will still be associated with mental health symptoms at a follow-up point.

## 2. Materials and Methods

This was a longitudinal study of mental health symptoms among residents of Nassau, Suffolk, Queens, and Richmond (Staten Island) counties in New York, which has been described previously [22,23,24]. Initial surveys were conducted 11 to 28 months after Hurricane Sandy at recruitment venues identified in conjunction with community and government partners. Participants were recruited using convenience-sampling techniques from community sites, including senior centers, libraries, gymnasiums, faith-based centers, community colleges, and community centers in both heavily and less affected areas across the region.

Out of the original 673 participants from the baseline survey, 130 subjects (19.3%) participated in the follow-up study, which relied on convenience-sampling techniques via phone, mail, and email recruitment from among the initial participants. One-year follow-up participants were reimbursed for their time with a $20 gift card.

Follow-up surveys were distributed via mail, telephone and email. Participants in the baseline study were called using the phone numbers provided during the initial survey. Voicemail messages were left for individuals who did not answer their phones, and persistent efforts were made to call each individual at different times of the day. Repeated emails were sent to all subjects who had provided their address and had not yet completed the research survey between February and April of 2016; five blasts were sent for each subject. The initial survey was self-administered, while the follow-up survey was self-administered when collected via mail or email, but was researcher-administered when collected via telephone. A small percentage of surveys were administered in person at a local retirement home. Of the 130 surveys, 12 (9.1%) were answered in person, 40 (30.8%) were answered via telephone, 48 (37.0%) were answered via postal mail, and 30 (23.1%) were answered via email.

### 2.1. Mental Health and Behavioral Outcomes

The primary outcomes measured were mental health symptoms of anxiety, depression and PTSD. Anxiety and depression symptoms were assessed using the previously validated Patient Health Questionnaire-4 (PHQ-4) [25]. Participants were categorized as having anxiety or depression symptoms if they scored a 2 or greater on questions relating to anxiety or depression. PTSD symptoms were assessed using the previously validated Civilian PTSD Questionnaire−Hurricane Sandy Specific (PTSD/PCL^-^S) [26]. Patients with a score of 30 or greater were categorized as having PTSD symptoms. The Cronbach’s alpha was 0.88 for baseline anxiety, 0.81 for follow-up anxiety, 0.86 for baseline depression, 0.78 for follow-up depression, 0.96 for baseline PTSD, and 0.95 for follow-up PTSD. Smoking and alcohol use were also asked about, and were secondary outcomes in the current study. Participants were categorized as smokers if they currently used tobacco and as problem drinkers if they exceeded the *National Institute on Alcohol Abuse and Alcoholism* (NIAAA) assessment guidelines (≥14 drinks/week in men; ≥7 women; ≥5 drinks on a single occasion in the past week in men; ≥4 women) [27].

### 2.2. Hurricane Exposure

Participants were asked at baseline to answer questions regarding their level of hurricane exposure and these questions were separated into three categories, consistent with the category classifications in Schwartz et al. [22]. Personal exposures dealt with exposures that directly affected the participant or their family, and property exposures related to the level of personal property affected and financial hardship experienced. A total of 16 personal exposures and 14 property exposures were measured; the level of exposure in each category was determined by taking the sum of the number of each kind of exposures an individual experienced, for a total possible score of 30 exposures. Participant total scores ranged between 0 and 21 (Table S1). The Cronbach’s alpha was 0.62 for baseline personal exposure and 0.85 for baseline property exposure. A reliability study showed that self-reported total exposure did not change significantly between baseline and one-year interviews (mean difference = 0.13, *p* = 0.34), although baseline exposure scores were used in analyses.

### 2.3. Covariates

Demographic information including age, sex, race, and education was also collected in the initial survey. Self-reported, physician-diagnosed mental health diagnoses (anxiety disorder, depression, PTSD, schizophrenia, bipolar, substance/alcohol abuse, substance/prescription abuse, or other mental health problems) were recorded during both the first and second survey waves. 

### 2.4. Statistical Analysis

Mental health and behavioral outcomes were treated as dichotomized variables using commonly employed clinical-relevant cutoffs in the main analysis to increase the interpretability of the results, while both dichotomized and continuous mental health outcomes were considered in explorative analyses. The prevalence of mental health symptoms at baseline and follow-up was analyzed using McNemar’s Test for dichotomous outcomes and Wilcoxon signed-rank tests for continuous outcomes due to non-normal distribution of these variables (Shapiro-Wilk test for baseline anxiety (*p* < 0.0001); follow-up anxiety (*p* < 0.0001); baseline depression (*p* < 0.0001); follow-up depression (*p* < 0.0001); baseline PTSD (*p* < 0.0001); follow-up PTSD (*p* < 0.0001). Determinants of mental health symptoms at follow-up were assessed using logistic and linear regression models adjusted for the following covariates: age, gender, ethnicity, education, presence of anxiety, depression, or PTSD symptoms at baseline, pre-existing physician-diagnosed mental health condition, time since Hurricane Sandy, and medical insurance status. As the main purpose of the study is to identify the longitudinal impact of hurricane exposure, the analysis was conducted on the 130 participants who had both baseline and follow-up data, excluding the 543 participants who only had baseline data. Chi-square and Wilcoxon-Mann-Whitney tests were used to test for differences between the 130 participants who completed the follow-up and the remaining 543. The majority of the variables were without missing data, with five variables having one to two missing data, which were treated as missing at random. All analyses were performed in SAS version 9.4 (SAS Institute Inc., Cary, NC, USA) and R Studio version 3.2.2 (R Foundation for Statistical Computing, Vienna, Austria).

## 3. Results

### 3.1. Characteristics of the Study Population

The majority of the 130 respondents were female (77.7%), white (57.0%), and had at least a high school diploma (94.6%) at baseline, while 65 participants (50%) met the criteria for anxiety, 46 (35.4%) for depression, and 37 (28.7%) for Hurricane Sandy-related PTSD symptoms (Table 1). Most subjects (n = 94; 72.3%) did not report having a history of diagnosed mental health disorders at baseline. Among those who reported a mental health diagnosis, 17 (13.1%) reported a diagnosis prior to, but not after Hurricane Sandy, 8 (6.2%) after Hurricane Sandy, and 11 (8.5%) both before and after the hurricane. On average (mean ± standard deviation), participants had 1.2 ± 1.64 personal exposure scores, 4.04 ± 3.41 property exposure scores and 5.28 ± 4.59 total exposure scores. 

The 130 participants who completed the follow-up study were partially representative of the 543 subjects who did not agree to answer follow-up questions about race/ethnicity (χ^2^ (3) = 3.48, *p* = 0.32), education status (χ^2^ (1) = 0.44, *p* = 0.51), medical insurance (χ^2^ (1) = 0.28, *p* = 0.60), and age (*p* = 0.17). The follow-up sample, however, was composed of a higher proportion of females (79% vs. 61%; χ^2^ (1) = 13.7, *p* < 0.001) and had higher total exposure (5.28 ± 4.59 vs. 3.56 ± 3.76, *p* < 0.001) compared to the original sample.

### 3.2. Changes in Mental Health Symptoms between Baseline and Follow-Up

The prevalence of participants with anxiety (50.0% to 41.5%), depression (35.4% to 30.8%), and PTSD (29.2% to 24.8%) symptoms decreased from baseline to follow-up (Table 2). There were statistically significant decreases in anxiety scores (mean difference = −0.33, *p* < 0.01) and PTSD scores (mean difference = −1.98, *p* = 0.001) between baseline and follow-up, but differences in depression scores were not statistically significant (mean difference = −0.12, *p* < 0.39). There were statistically significant correlations between mental health variables at baseline and at follow-up (Table S2). There was no significant association between demographic variables and changes in anxiety, depression, and PTSD scores between baseline and follow-up (data not shown).

### 3.3. Factors Associated with Mental Health Symptoms at Follow-Up

**Figure 1:**
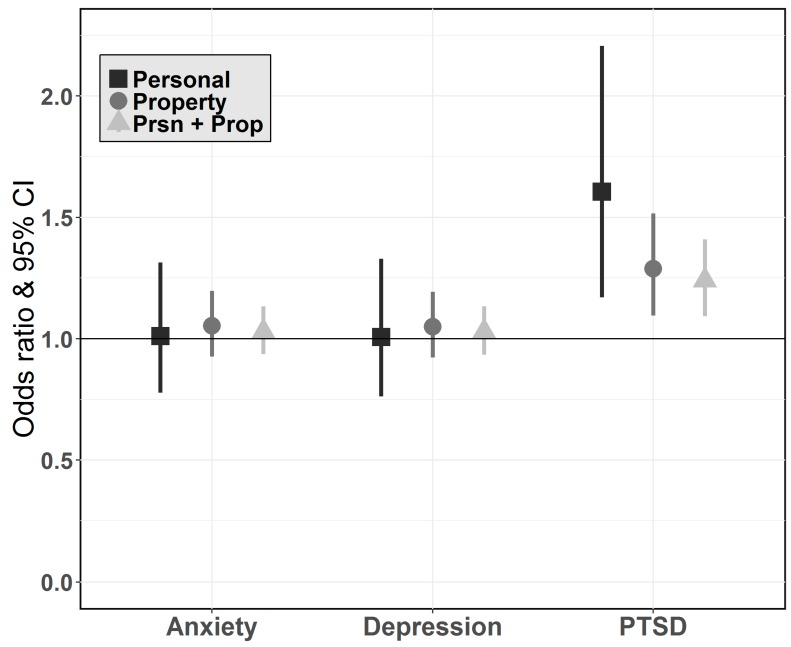
Association between hurricane exposure (personal, property, personal + property) and mental health at follow-up (anxiety, depression, PTSD symptoms). Models were adjusted for age, gender, race, education, medical insurance, existing mental health conditions, elapsed time between Hurricane Sandy and baseline, and mental health condition at baseline.

Experiencing personal damage (OR_adj_ 1.6, 95% CI [1.2–2.2]), property damage (OR_adj_ 1.3, 95% CI [1.1–1.5]), or a combination of personal and property damage (OR_adj_ 1.2, 95% CI [1.1–1.4]) was positively associated with PTSD symptoms, but not with anxiety or depression symptoms at follow-up (Figure 1). Having anxiety, depression, or PTSD at baseline was a strong predictor of having anxiety (OR_adj_ 2.8, 95% CI [1.1–6.8]), depression (OR_adj_ 7.4, 95% CI [2.3–24.1]) and PTSD (OR_adj_ 4.1, 95% CI [1.1–14.6]) symptoms at follow up. Female gender was positively associated with anxiety (OR_adj_ 3.4, 95% CI [1.1–10.9]), while having a history of mental health concerns prior to Hurricane Sandy was positively associated with PTSD symptoms (OR_adj_ 3.5, 95% CI [1.1–11.0]). When continuous measures of mental health outcomes were used in the models, total exposure was significantly associated with PTSD (β = 0.69, *p* = 0.001), but not anxiety (β = 0.01, *p* = 0.76) or depression (β = −0.02, *p* = 0.62) symptoms (Table 3).

Total exposure to Hurricane Sandy was not statistically associated with smoking (OR_adj_ 1.0, 95% CI [0.9–1.2]) or problem drinking (OR_adj_ 0.9, 95% CI [0.9–1.0]) after adjusting for age, gender, race, education, medical insurance, existing mental health conditions, elapsed time between Hurricane Sandy and baseline, and mental health condition at baseline.

## 4. Discussion

The current analysis indicates that property and personal hurricane exposures were associated with statistically significant increased odds of PTSD symptoms at follow-up, but not with anxiety or depression symptoms, suggesting that there may be a persistence of the negative impact of hurricanes on PTSD specifically. Previous studies following Hurricane Katrina and Hurricane Sandy have also noted that those exposed to greater stressors and property damage were more likely to demonstrate symptoms of PTSD and mental illness [9,28,29]. Similarly, there is evidence in the literature that the relationship between property exposure and PTSD can also be driven by secondary traumas that are associated with a loss of community and with searching for a new home [12,30]. This has been shown to have been more devastating among low-income individuals after Katrina [12] and Sandy [29], and is possible in this study as well, although we did not see differences by education or insurance status. Only one study has reported on post-traumatic stress at two time points following Sandy [29], and found that disaster-related stressors were significantly associated with higher post-traumatic stress at the first time point [29]. This work, however, sampled a cross-sectional, different set of participants at each time point, and thus was not longitudinal. Additionally, the current work relies on a more comprehensive definition of hurricane exposure, adding financial loss, access to medications, and vehicle loss, which are important determinants of mental health. As such, this work is the first to our knowledge to study longitudinal mental health symptoms within the same sample over time following Hurricane Sandy.

Previous cross-sectional and longitudinal studies of mental health outcomes after Katrina have reported that while a lack of social support does not strictly cause post-traumatic symptoms, a lack of social resources can increase the risk of stressors following a disaster, which in turn, influences post-traumatic stress [10,31,32,33,34].

These findings can also be placed into context with the Conservation of Resource theory (COR) [35,36], a framework to understand traumatic stress that has been applied following disasters [15,37,38,39,40]. Briefly, COR asserts that “individuals strive to obtain, retain, foster, and protect those things they centrally value”, termed resources, which universally include health, well-being, family, and also objects (cars, homes), conditions (employment, marriage), personal traits (self-efficacy, self-esteem), and energy (knowledge, money) [41]. Natural disasters subsequently threaten and destroy these resources and thus create stress. However, those with greater resources are less vulnerable to resource loss and can rebound more robustly compared to those with fewer resources that have less capacity to bounce back [41]. After a natural disaster, disparities exist between those with differing amounts of personal, social and material resources, informing long-term recovery after initial exposure to a natural disaster.

COR has emerged in analyses following Hurricane Katrina [42,43,44] and Hurricane Sandy [15,45], linking a loss of personal resources to the stress of displacement [42] and the loss of personal property, social support and physical health to long-term psychological distress [43]. However, the presence of personal and social resources can mitigate negative outcomes following exposure [45], but there are differences among people in their capacity to protect and regain resources that help mitigate the stress of hurricane exposure [15]. Although COR was not explicitly tested in this study, this work assessed object resources (property and personal exposure assessments), personal traits (personal exposure assessment) and energy resources (education status) and realized the association between larger initial personal and property exposures and the presence of long-term PTSD symptoms.

Presenting with symptoms of anxiety, depression, or PTSD at baseline were the strongest predictors of having anxiety, depression, or PTSD symptoms at follow-up across all three exposure types (personal, property, personal and property). Additionally, having a history of mental health concerns was a strong predictor of PTSD symptoms. This supports the literature stating that people with pre-existing mental health conditions and exposure to trauma were more vulnerable to these following Hurricane Katrina [46] and Hurricane Sandy [47]. Although average scores for anxiety and PTSD symptoms decreased between the initial and follow-up surveys, this does not reflect universal decreases in mental health symptoms for all participants, nor does it minimize the importance of the high prevalence of anxiety, depression, and PTSD symptoms at follow-up. As such, the results indicate the persistence of poor mental health outcomes in this sub-population exposed to Hurricane Sandy, and the need for future research to integrate measures of social support into mental health assessments in order to mitigate the long-term impacts of hurricane exposure.

There are several limitations to this study. This research relies on self-reported hurricane exposure items, which allows for recall bias, although it should be noted that no significant differences were found in the hurricane exposure assessment between baseline and follow-up, thus strengthening the validity of the exposure assessment. Furthermore, it is possible that people who were heavily affected by the hurricane or who were experiencing mental health difficulties were more prone to participate in research that could potentially mitigate their mental health symptoms, thereby potentially increasing the prevalence of mental health symptoms in the cohort. However, efforts were made to recruit participants from communities throughout the region, including those not highly affected by the hurricane, and from community events and organizations that were not in any way specific to either mental health service provision or Hurricane Sandy-related service provision. Also, as in prior studies, mental health symptoms were evaluated using self-reported measures that are not diagnostic in nature, but result in assessments of symptomatology. These measures, however, have repeatedly demonstrated validity and reliability and are consistently correlated with more comprehensive diagnostic tools assessing their corresponding mental health disorder and mental health symptoms [25,26,48,49,50]. Another limitation was the low response rate to the second wave of surveys. While efforts were made to increase the ease and accessibility of surveys for participants by providing the survey via email, the response rate was detrimentally impacted by the reality that participants declined to answer their phones. Additionally, participants that completed both waves of the survey were composed of more females and had higher levels of hurricane exposure compared to the remaining cohort that did not complete follow-up questionnaires. Despite the limitations, this study is the only one to date to use a population-based sample of people residing in Long Island, Staten Island and Queens with longitudinal follow-up after Hurricane Sandy. The contribution of longitudinal results may inform future long-term rescue and recovery efforts in the event of another natural disaster. 

## 5. Conclusions

In summary, there were significant decreases in anxiety and PTSD, but not depression scores, between baseline and follow-up. The strongest predictor of experiencing any mental health symptom after a disaster, however, was having anxiety, depression, or PTSD symptoms at baseline. Property, personal, and overall hurricane exposures were associated with an increased risk of having PTSD symptoms at follow-up, but significant effects were not observed between any type of hurricane exposure and anxiety or depression symptoms. To provide appropriate assistance and treatment after future natural disasters, mental health care providers and policymakers must consider the long-term effects of hurricane exposure on mental health, especially on PTSD, and must also focus on the vulnerable subgroup of those with existing mental health concerns.

## Figures and Tables

**Table 1:** Characteristics of the study population at baseline.

Variable		N	%
Sex	Male	29	22.31
	Female	101	77.69
Race/Ethnicity (missing n = 2)	White	73	57.03
	Black	25	19.53
	Hispanic Ethnicity	18	14.06
	Other/Mixed	12	9.38
Education (missing n = 1)	<High School	7	5.43
	≥High School	122	94.57
Medical Insurance (missing n = 1)	No	11	8.53
	Yes	118	91.47
Problem Alcohol Drinkers	No	98	75.38
	Yes	32	24.62
Current Smoker	No	116	89.23
	Yes	14	10.77
Mental health History	No	94	72.31
	Yes	36	27.69
Anxiety (PHQ4 score)	<2	65	50.00
	≥2	65	50.00
Depression (PHQ4 score)	<2	84	64.62
	≥2	46	35.38
Post-Traumatic Stress Disorder (PTSD) (PCL-S) (missing n = 1)	<30	92	71.32
	≥30	37	28.68
Mental Health problems	Never	94	72.31
	Before	17	13.08
	After	8	6.15
	Before and After	11	8.46
	Mean ± SD	Median	Range
Age (years, missing n = 1)	49.73 ± 20.82	52	18–92
Elapsed time between Sandy and Baseline questionnaire (months, missing n = 0)	14.47 ± 3.26	13.13	11.8–27.9
Personal damage score (missing n = 0)	1.25 ± 1.64	1	0–8
Property damage score (missing n = 0)	4.04 ± 3.41	3	0–14
Personal and propertydamage score (missing n = 0)	5.28 ± 4.59	3.5	0–21

**Table 2:** Prevalence of mental health symptoms at baseline and follow-up.

Variables		Baseline		Follow-Up		*p*-Value
		N	%	N	%	
Anxiety	<2	65	50.00	76	58.5	0.11^a^
	≥2	65	50.00	54	41.5	
	Mean ± SD	129	1.82 ± 1.84	129	1.49 ± 1.73	0.01^b^
Depression	<2	84	64.62	90	69.23	0.38 ^a^
	≥2	46	35.38	40	30.77	
	Mean ± SD	130	1.22 ± 1.72	130	1.10 ± 1.61	0.39 ^b^
PTSD	<30	92	71.32	97	75.19	0.44 ^a^
	≥30	37	58.68	32	24.81	
	Mean ± SD	129	26.95 ± 12.72	129	24.97 ± 11.57	0.001 ^b^

^a^ McNemar’s Test; ^b^ Wilcoxon Signed-Rank Test.

**Table 3:** Association between exposure and mental health symptoms.

	Anxiety		Depression		PTSD	
	Estimate	*p*-Value	Estimate	*p*-Value	Estimate	*p*-Value
**Total Exposure Model**						
Intercept	−0.80722	0.6603	0.79931	0.638	13.67105	0.2423
Mental Health Baseline	1.08977	0.0005	0.88404	0.0021	4.37012	0.0257
Gender	0.62258	0.0903	0.06906	0.8377	0.13119	0.9551
Age	−0.00334	0.6791	−0.00234	0.7529	0.05646	0.2685
Black	−0.48665	0.2135	0.21294	0.5538	4.84212	0.0513
Hispanic	0.03364	0.8798	−0.0386	0.8512	−0.35384	0.8027
Other/Mixed	0.11276	0.8265	1.24303	0.01	0.60231	0.8592
Education	0.11743	0.8531	−0.67738	0.2495	−6.26434	0.1211
Mental Health History	0.90104	0.0093	0.90403	0.0049	6.97867	0.0016
Medical Insurance	0.23852	0.6571	0.09397	0.85	0.06583	0.9852
Elapsed Time	0.01763	0.5895	0.02444	0.419	0.35813	0.0887
Total Exposure	0.00999	0.7626	−0.01537	0.6152	0.69631	0.0012
**Personal Exposure Model**						
Intercept	−0.66597	0.7154	0.79232	0.6389	15.93477	0.1785
Mental Health Baseline	1.10431	0.0004	0.87524	0.0022	4.94715	0.0126
Gender	0.65571	0.0736	0.07207	0.8299	0.56356	0.8111
Age	−0.00349	0.6658	−0.00286	0.7002	0.07215	0.1661
Black	−0.54218	0.1628	0.20651	0.5625	4.26012	0.0886
Hispanic	0.04301	0.8477	−0.01686	0.9351	−0.95971	0.5088
Other/Mixed	0.10969	0.8311	1.22454	0.0111	1.40813	0.683
Education	0.0987	0.8761	−0.66869	0.2542	−6.85456	0.095
Mental Health History	0.92534	0.0075	0.90824	0.0046	7.1842	0.0014
Medical Insurance	0.21566	0.6891	0.06753	0.8922	0.46097	0.8988
Elapsed Time	0.01663	0.611	0.02378	0.4316	0.37249	0.0819
Personal Exposure	−0.03755	0.6804	−0.05971	0.4779	1.54794	0.0094
**Property Exposure Model**						
Intercept	−0.88509	0.6296	0.76053	0.6547	13.87039	0.2381
Mental Health Baseline	1.07513	0.0006	0.8802	0.0023	4.28154	0.0301
Gender	0.60858	0.097	0.06018	0.8583	0.23397	0.9204
Age	−0.00365	0.6513	−0.00226	0.7618	0.04895	0.3407
Black	−0.464	0.2334	0.22806	0.5252	4.64593	0.0619
Hispanic	0.0457	0.8378	−0.04226	0.8382	−0.04199	0.9766
Other/Mixed	0.09979	0.8461	1.24465	0.01	0.33993	0.9208
Education	0.13396	0.8326	−0.67249	0.2536	−6.1866	0.1279
Mental Health History	0.89183	0.0099	0.89709	0.0052	7.08582	0.0014
Medical Insurance	0.2298	0.6685	0.10165	0.838	−0.33084	0.9261
Elapsed Time	0.01762	0.5891	0.02474	0.4135	0.347	0.1006
Property Exposure	0.02684	0.5448	−0.01349	0.7423	0.88476	0.0023

Models were adjusted for age, gender, race, education, medical insurance, existing mental health conditions, elapsed time between Hurricane Sandy and baseline, and mental health condition at baseline.

## Supplementary Materials

The following are available online at www.mdpi.com/1660-4601/14/9/957/s1, Table S1: Description of the exposure measurements collected, Table S2: Spearman correlation matrix of continuous study variables.
