# Supplementary material for: Longitudinal Impact of Hurricane Sandy Exposure on Mental Health Symptoms
Source: Int J Environ Res Public Health. 2017 Aug 24;14(9):957. doi: 10.3390/ijerph14090957 (PMC5615494; doi:10.3390/ijerph14090957)
Supplement: Supplementary file 1 [file ijerph-14-00957-s001.zip › Table_S2_proofed_final.docx]

| **Variables (n=124)** | Anxiety at baseline | Depression at baseline | PTSD at baseline | Anxiety at follow-up | Depression at follow-up | PTSD at follow-up | Age at follow-up | Elapsed Time (from exposure to follow-up) | Property exposure | Personal exposure |
| --- | --- | --- | --- | --- | --- | --- | --- | --- | --- | --- |
| Anxiety at baseline | 1 | 0.61462 | 0.49732 | 0.54466 | 0.45145 | 0.45268 | 0.09609 | 0.10362 | 0.25715 | 0.1187 |
| p-value | - | <.0001 | <.0001 | <.0001 | <.0001 | <.0001 | 0.2884 | 0.2521 | 0.0039 | 0.1892 |
| Depression at baseline | 0.61462 | 1 | 0.47675 | 0.5286 | 0.54359 | 0.4606 | -0.15684 | 0.15016 | 0.19487 | 0.15532 |
| p-value | <.0001 | - | <.0001 | <.0001 | <.0001 | <.0001 | 0.0819 | 0.096 | 0.0301 | 0.085 |
| PTSD at baseline | 0.49732 | 0.47675 | 1 | 0.38179 | 0.3466 | 0.63436 | 0.13152 | -0.11366 | 0.47923 | 0.3699 |
| p-value | <.0001 | <.0001 | - | <.0001 | <.0001 | <.0001 | 0.1454 | 0.2088 | <.0001 | <.0001 |
| Anxiety at follow-up | 0.54466 | 0.5286 | 0.38179 | 1 | 0.73391 | 0.56167 | -0.17035 | 0.16308 | 0.21071 | 0.07666 |
| p-value | <.0001 | <.0001 | <.0001 | - | <.0001 | <.0001 | 0.0585 | 0.0703 | 0.0188 | 0.3974 |
| Depression at follow-up | 0.45145 | 0.54359 | 0.3466 | 0.73391 | 1 | 0.53578 | -0.19366 | 0.1577 | 0.11524 | 0.05304 |
| p-value | <.0001 | <.0001 | <.0001 | <.0001 | - | <.0001 | 0.0311 | 0.0802 | 0.2025 | 0.5585 |
| PTSD at follow-up | 0.45268 | 0.4606 | 0.63436 | 0.56167 | 0.53578 | 1 | 0.04384 | 0.08931 | 0.30865 | 0.25915 |
| p-value | <.0001 | <.0001 | <.0001 | <.0001 | <.0001 | - | 0.6288 | 0.3239 | 0.0005 | 0.0037 |
| Age at follow-up | -0.09609 | -0.15684 | 0.13152 | -0.17035 | -0.19366 | 0.04384 | 1 | -0.49004 | 0.07293 | -0.05896 |
| p-value | 0.2884 | 0.0819 | 0.1454 | 0.0585 | 0.0311 | 0.6288 | - | <.0001 | 0.4208 | 0.5154 |
| Elapsed Time (from exposure to follow-up | 0.10362 | 0.15016 | -0.11366 | 0.16308 | 0.1577 | 0.08931 | -0.49004 | 1 | -0.04308 | 0.04924 |
| p-value | 0.2521 | 0.096 | 0.2088 | 0.0703 | 0.0802 | 0.3239 | <.0001 | - | 0.6348 | 0.5871 |
| Property exposure | 0.25715 | 0.19487 | 0.47923 | 0.21071 | 0.11524 | 0.30865 | 0.07293 | -0.04308 | 1 | 0.58878 |
| p-value | 0.0039 | 0.0301 | <.0001 | 0.0188 | 0.2025 | 0.0005 | 0.4208 | 0.6348 | - | <.0001 |
| Personal exposure | 0.1187 | 0.15532 | 0.3699 | 0.07666 | 0.05304 | 0.25915 | -0.05896 | 0.04924 | 0.58878 | 1 |
| p-value | 0.1892 | 0.085 | <.0001 | 0.3974 | 0.5585 | 0.0037 | 0.5154 | 0.5871 | <.0001 | - |

Table S2. Spearman correlation matrix of continuous study variables.
