# Supplementary material for: Longitudinal Impact of Hurricane Sandy Exposure on Mental Health Symptoms
Source: Int J Environ Res Public Health. 2017 Aug 24;14(9):957. doi: 10.3390/ijerph14090957 (PMC5615494; doi:10.3390/ijerph14090957)
Supplement: Supplementary file 1 [file ijerph-14-00957-s001.zip › Table_S1_proofed_final.docx]

Table S1. Description of the exposure measurements collected.

| **Total Exposure (total score range 0-30)** | |
| --- | --- |
| **Personal Exposure (score range 0-16)** | **Property Exposure (score range 0-14)** |
| -Family member missing | -Loss of power/electricity |
| -Family member died | -Displaced from home |
| -Friend missing | -Home damaged or destroyed |
| -Friend died | -Business damaged or destroyed |
| -You were physically harmed | -Vehicle loss |
| -Someone in your family was physically harmed | -Major property loss other than home (ex., furniture) |
| -Friend was physically harmed | -Disaster resulted in unemployment (self) |
| -Your life was in danger | -Disaster resulted in unemployment (household) |
| -The life of family member(s) in danger | -Unable to access necessary medical care |
| -Evacuated quickly with no time to -prepare | -Unable to access gasoline for vehicle |
| -Prolonged separation from family | -Significant financial loss |
| -Pet missing or dead | -Great difficulty accessing food for self/family |
| -Witnessed death/injury (self or household) | -Great difficulty commuting to work/school |
| -Witnessed death/injury (other; ex. neighbor) | -Did you experience flooding in your home caused by Hurricane Sandy |
| -Assisted with rescue/recovery (self or household) |  |
| -Assisted with rescue/recovery (other; ex. neighbor) |  |
